# Association Between Perceived Medical Errors and Suicidal Ideation Among Chinese Medical Staff: The Mediating Effect of Depressive Symptoms

**DOI:** 10.3389/fmed.2022.807006

**Published:** 2022-02-10

**Authors:** Zhen Wei, Yifan Wang, Shijun Yang, Long Sun

**Affiliations:** ^1^Centre for Health Management and Policy Research, School of Public Health, Cheeloo College of Medicine, Shandong University, Jinan, China; ^2^NHC Key Lab of Health Economics and Policy Research, Shandong University, Jinan, China

**Keywords:** perceived medical errors, suicidal ideation, depressive symptoms, medical staff, cross-sectional study

## Abstract

Both medical errors and suicidal behaviors are important problems for medical staff. Although the association between them was implied in previous studies, their association has not been built until now. In this study, the first aim was to build the association between perceived medical errors and suicidal ideation, and we also want to explore the mediating role of depression in the association between perceived medical errors and suicidal ideation among Chinese medical staff. In this study, we interviewed 3,338 medical staff in Chinese general hospitals. Questions about suicidal ideation (SI) and perceived medical errors were interviewed for medical staff. Depressive symptoms were evaluated by the Chinese version of Center for Epidemiologic Studies Depression Scale (CES-D). Social-demographic and occupation-related variables were also assessed in the data collection. This study found that the prevalence of suicidal ideation among medical staff was 9%. After the adjustment for controlling variables, suicidal ideation was significantly associated with a higher level of depression (OR = 1.10, *p* < 0.001) and perceived medical errors (OR = 2.41, *p* < 0.001). The other associated factors were female (OR = 2.21, *p* < 0.001), religious belief (OR = 2.66, *p* < 0.001), and weekly work hours (OR = 1.02 *p* < 0.001). The mediating effect of depressive symptoms on the association between perceived medical errors and suicidal ideation was also supported in this study, and it can explain the 38.73% of the total effects of the perceived medical errors on suicidal ideation. The medical staff, with perceived medical errors, were in higher risk of suicidal ideation, and depressive symptoms can partially mediate the association between perceived medical errors and suicidal ideation. For the medical staff who are experiencing medical errors, some scanning on their suicidal ideation and depressive symptoms are necessary to promote their mental health.

## Introduction

According to World Health Organization (WHO) global health estimates, the global age-standardized suicide rate was about 9 per 100,000 population in 2019, accounting for 1.3% of all deaths, which stated that suicide remained a leading cause of deaths worldwide (1). In all the global suicide cases, there were about 22 percent, which was accounted from China (2, 3). In the other sides, medical staff, as a special population with intense and stressful working environment, were also in higher risk of suicidal behaviors in China and other countries in the world (4, 5). In China, medical staff are also characterized by longer working hours, less social support, and more psychological strains (6, 7). Considering the less percentage of medical staff among populations (8), we have enough reasons to believe that Chinese medical staff may be in higher risk of psychological problems and suicide. Consequently, suicidal behaviors among medical staff were an important public health and societal problem in the world, especially in China.

Medical errors refer to mistakes committed by medical staff, which result in harm to the patients (9). According to the prior studies, there were about 210,000 to 400,000 deaths associated with medical errors worldwide each year (10), medical errors can damage the quality of life of the patients (11), and previous studies estimated that the cost of medical errors was about billions of dollars (12). In China, the previous studies also found that medical errors caused serious medical liabilities and economic burdens (13). However, it was strange that the burden and impact of medical errors on patients and society had been reported in many former studies. There were only a few studies that explored the impact of medical errors on medical staff themselves in previous years (14). Actually, medical staff who experiences medical errors may also be in a condition of psychological and emotional pain, and they also need to be helped (15).

In recent decades, there have been studies found that medical staff who perceived medical errors would experience a high level of self-blame, humiliation, guilt, negative self-perception, depression, and so on (16, 17). There were studies that supported that medical staff were in higher risk of negative emotional and psychological problems compared with non-medical staff (18, 19). Based on these two findings, we can assume that the medical staff who perceived medical errors may be in a very high risk of negative psychological problems in our society, which should be paid more attention. Furthermore, considering the identified association between negative psychological problems and suicidal behaviors (6, 20), we can also easily assume that the medical staff who perceived medical errors are also in higher risk of suicidal behaviors. In conclusion, these findings imply to us that the medical staff who perceived medical errors were in a higher risk of negative psychological problems, which further contribute to suicidal behaviors. In other words, negative psychological problems may mediate the association between perceived medical errors and suicidal behaviors.

Suicidal ideation (SI), one kind of suicidal behaviors, has been found to be a widespread and intensive predictor for suicide death, and most suicides occur within the year of the onset of suicidal ideation (21). Therefore, suicidal ideation, as a robust predictor of suicide, warrants attention (22, 23). For medical staff, although their high suicidal ideation rates had been found in prior studies (24), the association between perceived medical errors and suicidal ideation has not been tested until now, and the effect of depressive symptoms on this association was also not explored in previous studies.

To bridge this gap, with the exception of the aforementioned, the first aim for this study was to build the association between perceived medical errors and suicidal ideation among Chinese medical staff. Based on the previous findings, we also want to test the mediating effect of depressive symptoms on the association between perceived medical errors and suicidal ideation among the Chinese medical staff. This study provides evidence for improving mental health and reducing suicidal ideation among medical staff, and for promoting medical staff to provide better health care services.

## Methods

### Study Design and Participants

**Figure 1:**
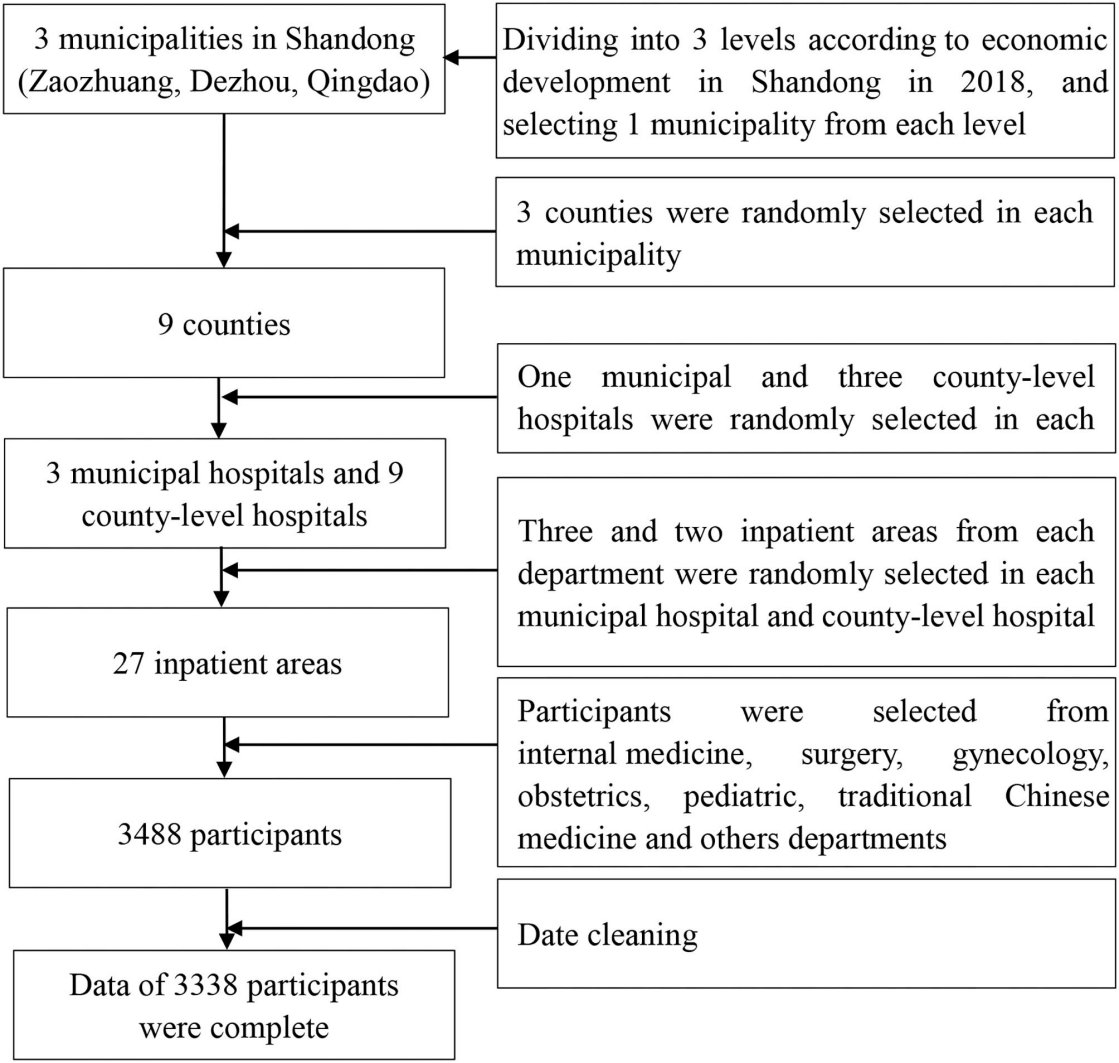
A flowchart of the sampling procedure.

In this study, we collected 3,338 valid questionnaires based on a cross-sectional design among medical staff in Shandong province, China, Shandong Province located in the east of China. It ranked second in the population (25), and the number of medical staff ranked first in China (26). In order to get the description of medical staff in Shandong, the multi-stratified random cluster sampling method was applied by the following steps. First, we divided all the 16 cities in Shandong province into three levels according to the Gross Domestic Product (GDP) *per capita* in 2018 (27), and one city was randomly selected from each level of GDP *per capita* (Zaozhuang, Dezhou, Qingdao). Second, one municipal hospital was randomly selected from all the municipal hospitals in each city. Third, three counties/districts were randomly selected in each city. In each county/district, one general hospital was randomly chosen. Therefore, a total of 12 hospitals were selected in 3 cities, including 3 municipal hospitals and 9 county-level hospitals. Fourth, three and two inpatient areas from each department were randomly selected in each municipal hospital and county-level hospital, respectively. Medical staff working on the survey date were asked to fill the questionnaires in the study. Finally, we collected 3,338 valid questionnaires with a 95.70% valid response rate (3,338/3,488). The flowchart of the sampling procedure is shown in Figure 1.

### Data Collection

This study was conducted between December 2018 and January 2019. Data were collected by the filed self-filling method. Well-trained investigators were responsible for distributing questionnaires to each respondent, interpreting questions, and checking returned questionnaires. Before the research began, we had communicated the aim of this study with medical staff and informed them that their participation would not be paid. Medical staff who were reluctant to cooperate with the interviewer were excluded in order to better obtain complete and accurate data. The study protocol was approved by the ethics committee of the school of public health, Shandong University, before the data collection (Ref. No. 20181219). Written informed consent was collected from all the participants.

### Measures

#### Suicidal Ideation

Suicidal ideation was measured by a single-item question, which was “Have you ever seriously considered suicide?” The answer could be chosen from yes (1) or no (0). Thus, the lifetime suicidal ideation was collected in this study. This question was widely used to evaluate the lifetime suicidal ideation worldwide, such as the US National Comorbidity Survey (21), and so on (28, 29).

#### Perceived Medical Error

Perceived medical errors were evaluated by the question “Have you made any medical errors in the past year?” The answer can also be chosen from yes (1) or no (0). This assessment method was based on prior similar studies (30–32), and the aim was to identify the events that were internalized as a medical error.

#### Depressive Symptoms

Depressive symptoms were measured by the Chinese version of Center for Epidemiologic Studies Depression (CES-D) scale, which is a widely accepted depressive symptoms assessment scale with good reliability and validity in the world (33, 34). The Chinese version of CES-D also proved a good reliability and validity in previous studies (35). In this study, the Cronbach's alpha of CES-D was 0.852.

#### Sociodemographic Variables

Gender was coded as male (0) and female (1). Age was calculated by subtracting the date of birth from the date of the survey. Marital status can be chosen from not married, married, divorced, widowed, and others. As there were most participants who are married, we recoded it into single (0) and married (1). Religious belief was evaluated by Buddhism, Taoism, Christianism, Catholicism, and others. As there were few medical staff with religious belief, we recoded it into yes (1) and no (0). Education was recoded into “0 = junior college and below” [including middle school and below, high school, technical secondary school, and junior college], and “1 = bachelor's degree and above” [including university, master, and doctor].

#### Occupation-Related Variables

Professions were estimated by clinicians, nurses, and medical technicians. Formal workers were evaluated by yes, no, and unclear, as only 1.1% of medical workers did not know whether they were formal workers or not. Formal worker was recoded as “0 = no” [including no and unclear], and “1 = yes.” Departmental head was measured as “0 = no,” and “yes = 1.” Hospital levels were measured by Level 3 and Level 2, which were mainly evaluated based on the scale, and the technical level of the hospitals by National Health Commission of China. It was recoded as “0 = Level 2,” and “1= Level 3.” Weekly working hours were a continuous variable.

### Statistical Analyses

All data were analyzed using IBM SPSS Statistics (web version 24.0). The *t*-tests were performed for continuous variables, and Chi-Square tests were performed for categorical variables. Binary logistic regressions were conducted to determine the potential association between perceived medical errors and suicidal ideation among medical staff. Then, SPSS macro program PROCESS V3.5 was used to verify the mediating effect of depressive symptoms on the association between perceived medical errors and suicidal ideation (36). All *P*-values were two-tailed and *p*-values < 0.05 were considered as statistically significant.

## Results

### The Characteristics of the Participants and Univariate Analysis for the Associated Factors of Suicidal Ideation

A total of 3,338 medical staff were included in this study, and their characteristics are presented in Table 1. In the sample, the females outnumbered the males, comprising 73.3% of the participants. Mean age of the respondents was 35.06 years with an SD of 8.38. The mean total scores of weekly work hours and depressive symptoms were 47.62 ± 9.46 and 14.63 ± 10.37, respectively. The prevalence of suicidal ideation was 9%. Regarding the medical errors, 2.8% of medical staff reported that they perceived medical errors. A more detailed information is demonstrated in Table 1.

**Table 1:** Characteristics and univariate analysis of suicidal ideation among medical staff.

**Characteristics**	**$\bar{\text{X}}$S/n(%)**	**Suicide ideation [*****n*** **(%)]**		**t/χ^2^**
		**Yes**	**No**	
Total	3,338 (100.0)	301 (9.0)	3,037 (91.0)	-
Gender				4.99^*^
Male	891 (26.7)	64 (7.2)	827 (92.8)	
Female	2,447 (73.3)	237 (9.7)	2,210 (90.3)	
Age (years)	35.06 ± 8.38	34.27 ± 7.16	35.14 ± 8.50	1.97
Marital status				4.51
Single	604 (18.1)	68 (11.3)	536 (88.7)	
Married	2,734 (81.9)	233 (8.5)	2,501 (91.5)	
Religious belief				22.45^***^
No	3,234 (96.9)	278 (8.6)	2,956 (91.4)	
Yes	104 (3.1)	23 (22.1)	81 (77.9)	
Degree of education				1.93
Junior college and below	429 (12.9)	31 (7.2)	398 (92.8)	
Bachelor degree and above	2909 (87.1)	270 (9.3)	2639 (90.7)	
Profession				0.12
Clinician	1,225 (36.7)	108 (8.8)	1,117 (91.2)	
Nurse	1,663 (49.8)	151 (9.1)	1,512 (90.9)	
Medical technician	450 (13.5)	42 (9.3)	408 (90.7)	
Formal worker				1.54
No	1,407 (42.2)	137 (9.7)	1,270 (90.3)	
Yes	1,931 (57.8)	164 (8.5)	1,767 (91.5)	
Departmental head				0.35
No	2,662 (79.7)	244 (9.2)	2,418 (90.8)	
Yes	676 (20.3)	57 (8.4)	619 (91.6)	
Hospital level				1.99
Level 3	1,448 (43.4)	119 (8.2)	1,329 (91.8)	
Level 2	1,890 (56.6)	182 (9.6)	1,708 (90.4)	
Weekly work hours	47.62 ± 9.46	49.74 ± 10.84	47.41 ± 9.29	−3.605^***^
Perceived medical errors				32.15^***^
No	3,244 (97.2)	277 (8.5)	2,967 (91.5)	
Yes	94 (2.8)	24 (25.5)	70 (74.5)	
Depressive symptoms	14.63 ± 10.37	24.86 ± 11.68	13.62 ± 9.66	−16.15^***^

* *p < 0.05*;

*** *p < 0.001*.

Table 1 also presented the results of the univariate analysis that tested the association between socio-demographic variables, occupation-related variables, perceived medical errors, depressive symptoms, and suicidal ideation. The results showed that the factors significantly associated with suicidal ideation were gender (χ^2^ = 4.99, *p* = 0.026), marital status (χ^2^ = 4.51, *p* = 0.034), religious belief (χ^2^ = 22.45, *p* < 0.001), weekly work hours (t = −3.605, *p* < 0.001), medical errors (χ^2^ = 32.15, *p* < 0.001), and depressive symptoms (t = −16.15, *p* < 0.001).

### Binary Logistic Regression Analysis Results of Suicidal Ideation Among Medical Staff

Logistic regression was conducted to explore the association between perceived medical errors and suicidal ideation. In Model A, we could find that the perceived medical errors were significantly associated with suicidal ideation (OR = 3.56, *p* < 0.001) after controlling the socio-demographic and occupation-related variables. In Model B, we further controlled depressive symptoms in the regression; the association between perceived medical errors and suicidal ideation was also significant (OR = 2.41, *p* < 0.001) in the regression. The other related variables were female (OR = 2.21, *p* < 0.001), religious belief (OR = 2.66, *p* < 0.001), and weekly work hours (OR = 1.02, *p* < 0.001). The detailed information is shown in Table 2.

**Table 2:** Logistic analysis for the factors associated with suicidal ideation among medical staff.

**Variables**	**Model A [OR (95% CI)]**	**Model B [OR (95% CI)]**
Gender (Ref. = Male)		
Female	1.70 (1.22, 2.36)^**^	2.21 (1.55, 3.16)^***^
Age (years)	1.00 (0.98, 1.02)	1.00 (0.98, 1.02)
Marital status (Ref. = Others)		
Married	0.78 (0.57, 1.08)	0.82 (0.58, 1.15)
Religious belief (Ref. = No)		
Yes	3.07 (1.88, 5.02)^***^	2.66 (1.55, 4.54)^***^
Degree of education (Ref. = Junior college and below)		
College and above	1.38 (0.91, 2.07)	1.37 (0.89, 2.11)
Profession (Ref.= Clinician)		
Nurse	0.96 (0.69, 1.35)	0.87 (0.60, 1.25)
Medical technician	1.25 (0.84, 1.88)	1.17 (0.76, 1.80)
Formal worker (Ref. = No)		
Yes	0.91 (0.68, 1.24)	0.91 (0.66, 1.26)
Departmental head (Ref. = No)		
Yes	0.96 (0.69, 1.34)	1.08 (0.76, 1.54)
Hospital level (Ref. = Level 3)		
Level 2	1.10 (0.85, 1.41)	0.96 (0.74, 1.26)
Weekly work hours	1.03 (1.02, 1.04)^***^	1.02 (1.01, 1.04)^***^
Perceived medical errors (Ref. = No)		
Yes	3.56 (2.18, 5.81)^***^	2.41 (1.43, 4.08)^***^
Depressive symptoms	–	1.10 (1.08, 1.11)^***^

^*^p < 0.05;

** *p < 0.01*;

*** *p < 0.001. OR, odd ratio; CI, confidence interval*.

### The Mediating Effect of Depressive Symptoms on the Association Between Medical Errors and Suicidal Ideation

Finally, to test the hypothesized mediation of depressive symptoms on the association between perceived medical errors and suicidal ideation, we found that all the direct effects, indirect effects, and total effects of perceived medical errors were significant. Thus, we could conclude that depressive symptoms played a partial mediating effect on the association between perceived medical errors and suicidal ideation, and the mediating effect of depression could explain the 38.73% of the total effect of perceived medical errors on suicidal ideation. The detailed information is shown in Table 3.

**Table 3:** Mediation of perceived medical errors and suicidal ideation by depressive symptoms.

**Path**	**Independent variable**	**Dependent variable**	**β**	**SE**	***p***
a	Perceived medical errors	Depressive symptoms	6.12	1.08	<0.001
b	Depressive symptoms	Suicidal ideation	0.09	0.27	<0.05
c'	Perceived medical errors	Suicidal ideation	0.88	0.01	<0.001

*SE, standard error*.

## Discussion

In this study, the results revealed that the prevalence of suicidal ideation among medical staff was 9%. We also sought to better understand the association between perceived medical errors and suicidal ideation among medical staff. As predicted, when we collectively examined our data, we found that perceived medical errors were significantly associated with suicidal ideation, and depressive symptoms have significantly and partially mediated the correlation between perceived medical errors and suicidal ideation.

Previous studies found that medical staff were in higher risk of suicide than general population (37), and the suicide rates among medical staff have been on the rise in the recent years (38). When we compared our results with prior studies, the prevalence of suicidal ideation in our study was roughly similar with other studies that were conducted among medical staff (39). Possible reasons may be that medical staff are at an increased occupational-specific risk about workplace violence, burnout, and medical errors, which can heighten the suicidal ideation among medical staff (40, 41).

Our results demonstrated that perceived medical errors were strongly correlated with suicidal ideation; medical staff who perceived medical errors were more likely to experience suicide ideation (41). As we know, suicidal ideation results from an interplay of psychological and societal factors (42). When medical staff were involved in medical errors, most of them might experience mood problems, but the medical organizational resources had insufficiently supported the medical staff after medical errors (43), and they may also experience discrimination in medical qualification and professional advancement from patients and hospitals. Both mood problems and discrimination were risk factors in suicidal ideation, which had been identified in previous studies (44).

This study also demonstrated that perceived medical errors were significantly and positively associated with depressive symptoms. Medical staff who perceived medical errors were more likely to report a higher risk of depression. This finding was consistent with previous study (45), which is the perceived medical errors were associated with roughly doubling in risk of depression. The possible explanation for this finding was that, when medical staff perceived medical errors, majority of them will attribute responsibilities for themselves (9). In addition to external punishment and blame, a series of personal and emotional reactions such as self-accusation, negative self-evaluation, shame, guilt, and so on will lead medical staff to experience depressive symptoms (43, 46).

The main finding in this study was the significant mediating effect of depressive symptoms on the association between perceived medical errors and suicidal ideation, and perceived medical errors had an indirect association with suicidal ideation largely through the effect of depression. In other words, the subsequent impact of perceived medical errors has increased the depression of the medical staff, which would result in suicidal ideation. Actually, the mechanism underlying the correlations among medical errors, depressive symptoms, and suicidal ideation had been discussed in the previous paragraphs. Medical errors can be viewed as adverse and negative events that occur in the delivery of health care services (47, 48). Previous studies showed that depressive symptoms play a mediated role in the association between untoward events and suicide risk (49–51). Consequently, when medical staff caused adverse events at work, the effect of perceived medical errors on suicidal ideation may work, in part, through depression.

In this study, we also found that female medical staff were more likely to experience suicidal ideation than the male medical staff, which was consistent with other studies among other populations (52). Previous studies found that male suicide rates were higher than females (53), but the prevalence of suicidal ideation among females was higher than males (5). Additionally, this study found that religious beliefs were risk factors in suicidal ideation, contrary to some previous studies (54); moreover, previous studies have also shown that religion does not necessarily protect against suicidal ideation, but it does protect against suicide attempts (55). Longer weekly work hours have also proven to be positively associated with suicidal ideation (6). The longer work hours, the higher risk of suicidal ideation. The reasons may be that longer work hours can lead to burnout and increased job stress, and lower quality of life, which are the strong predictors of suicidal ideation (56).

A considerable advantage of the current study was exploring the association between perceived medical errors, depressive symptoms, and suicidal ideation. As far as our information goes, this study is the first study to examine the mediating role of depressive symptoms in the association between perceived medical errors and suicidal ideation. Another advantage is that this study is based on the fact that previous studies have focused less on suicide issues among Chinese medical staff (24).

There are several limitations that should be considered. First, the cross-sectional design of this study and the different time frames for suicidal ideation and medical error limit the ability to determine the causal relationship between perceived medical errors, depressive symptoms, and suicidal ideation. A longitudinal design with the incident time of suicidal ideation and medical error will be helpful for us to understand their relationships. Second, the major variables in the current study were measured by self-report, which might lead to a recall bias. Third, we verified only one mediation variable in the current study; a more potential mechanism between perceived medical errors and suicidal ideation needs further exploration. Lastly, medical staff were all from general hospitals; a great caution should be exercised in generalizing the results in all kinds of hospitals in China.

Our findings indicated that perceived medical errors were both directly associated with suicidal ideation and indirectly related to this result through depressive symptoms. These findings have implication for the prevention of suicidal ideation among medical staff. On one hand, given the close relationship between depressive symptoms and suicidal ideation, preventive efforts should aim those with depressive symptoms, including but not limited to medical staff with medical errors. On the other hand, selectively targeting those with medical errors may be a useful strategy for reducing depressive symptoms among medical staff, which is a preventive method that is considerably upstream from suicidal ideation.

## Data Availability Statement

The raw data supporting the conclusions of this article will be made available by the authors, without undue reservation.

## Ethics Statement

The studies involving human participants were reviewed and approved by the Ethics Committee of School of Public Health, Shandong University. The patients/participants provided their written informed consent to participate in this study.

## Author Contributions

ZW wrote the original draft and analyzed the data. YW and SY collected the data. LS conceptualized the study and reviewed this manuscript. All the authors read and approved the final manuscript.

## Funding

The research was supported by the National Natural Science Foundation of China (71603149 and 71974114), Shandong Provincial Natural Science Foundation, China (ZR2016HQ01).

## Conflict of Interest

The authors declare that the research was conducted in the absence of any commercial or financial relationships that could be construed as a potential conflict of interest.

## Publisher's Note

All claims expressed in this article are solely those of the authors and do not necessarily represent those of their affiliated organizations, or those of the publisher, the editors and the reviewers. Any product that may be evaluated in this article, or claim that may be made by its manufacturer, is not guaranteed or endorsed by the publisher.

## References

[1] Oganization WH. Suicide Worldwide in 2019. Global Health Estimates. (2021).

[2] Hvistendahl M. Public health. Making sense of a senseless act. Science. (2012) 338:1025–7. 10.1126/science.338.6110.1025 23180841

[3] Zhang J Sun L Liu Y Zhang J. The change in suicide rates between 2002 and 2011 in China. Suicide Life Threat Behav. (2014) 44:560–8. 10.1111/sltb.12090 24690079

[4] Schernhammer E. Taking their own lives - The high rate of physician suicide. New Engl J Med. (2005) 352:2473–6. 10.1056/NEJMp058014 15958803

[5] Schernhammer ES Colditz GA. Suicide rates among physicians: a quantitative and gender assessment (meta-analysis). Am J Psychiatry. (2004) 161:2295–302. 10.1176/appi.ajp.161.12.2295 15569903

[6] Liu Y Zhang J Hennessy DA Zhao S Ji H. Psychological strains, depressive symptoms, and suicidal ideation among medical and non-medical staff in urban china. J Affect Disorders. (2019) 245:22–7. 10.1016/j.jad.2018.10.111 30366234

[7] Zhao S Zhang J Liu Y Ji H Lew B. The association between psychological strains and life satisfaction: Evidence from medical staff in China. J Affect Disord. (2020) 260:105–10. 10.1016/j.jad.2019.09.006 31494361

[8] Anand S Fan VY Zhang J Zhang L Ke Y Dong Z. China's human resources for health: quantity, quality, and distribution. Lancet. (2008) 372:1774–81. 10.1016/S0140-6736(08)61363-X 18930528

[9] Shanafelt TD Balch CM Bechamps G Russell T Dyrbye L Satele D. Burnout and medical errors among American surgeons. Ann Surg. (2010) 251:995–1000. 10.1097/SLA.0b013e3181bfdab3 19934755

[10] James JT. A new, evidence-based estimate of patient harms associated with hospital care. J Patient Saf. (2013) 9:122–8. 10.1097/PTS.0b013e3182948a69 23860193

[11] Weingart SN Wilson RM Gibberd RW Harrison B. Epidemiology of medical error. BMJ (Clinical research ed). (2000) 320:774–7. 10.1136/bmj.320.7237.774 10720365 PMC1117772

[12] Van Den Bos J Rustagi K Gray T Halford M Ziemkiewicz E Shreve J. The $17.1 billion problem: the annual cost of measurable medical errors. Health Affairs. (2011) 30:596–603. 10.1377/hlthaff.2011.0084 21471478

[13] Li H Dong S Liao Z Yao Y Yuan S Cui Y. Retrospective analysis of medical malpractice claims in tertiary hospitals of China: the view from patient safety. BMJ Open. (2020) 10:e034681. 10.1136/bmjopen-2019-034681 32973050 PMC7517568

[14] Ozeke O Ozeke V Coskun O Budakoglu II. Second victims in health care: current perspectives. Adv Med Educ Pract. (2019) 10:593–603. 10.2147/AMEP.S185912 31496861 PMC6697646

[15] Wu AW. Medical error: the second victim: the doctor who makes the mistake needs help too. BMJ. (2000) 320:726–7. 10.1136/bmj.320.7237.726 10720336 PMC1117748

[16] Pratt SD Jachna BR. Care of the clinician after an adverse event. Int J Obstet Anesth. (2015) 24:54–63. 10.1016/j.ijoa.2014.10.001 25499810

[17] Kwok C. Depression, stress, and perceived medical errors in singapore psychiatry residents. Acad Psychiatry. (2021) 45:169–73. 10.1007/s40596-020-01376-w 33409942

[18] Gold KJ Sen A Schwenk TL. Details on suicide among US physicians: data from the National Violent Death Reporting System. Gen Hosp Psychiatry. (2013) 35:45–9. 10.1016/j.genhosppsych.2012.08.005 23123101 PMC3549025

[19] Dyrbye LN Thomas MR Shanafelt TD. Systematic review of depression, anxiety, and other indicators of psychological distress among US and Canadian medical students. Acad Med. (2006) 81:354–73. 10.1097/00001888-200604000-00009 16565188

[20] Pereira-Lima K Mata DA Loureiro SR Crippa JA Bolsoni LM Sen S. Association between physician depressive symptoms and medical errors: a systematic review and meta-analysis. JAMA network open. (2019) 2:e1916097. 10.1001/jamanetworkopen.2019.16097 31774520 PMC6902829

[21] Kessler RC Borges G Walters EE. Prevalence of and risk factors for lifetime suicide attempts in the National Comorbidity Survey. Arch Gen Psychiatry. (1999) 56:617–26. 10.1001/archpsyc.56.7.617 10401507

[22] Klonsky ED May AM. The three-step theory (3ST): A New Theory of Suicide Rooted in the “Ideation-to-Action” Framework. Int J Cogn Ther. (2015) 8:114–29. 10.1521/ijct.2015.8.2.114

[23] Harmer B Lee S Duong TVH Saadabadi A. Suicidal Ideation. StatPearls Treasure Island (FL). (2021).

[24] Dutheil F Aubert C Pereira B Dambrun M Moustafa F Mermillod M. Suicide among physicians and health-care workers: A systematic review and meta-analysis. PLoS ONE. (2019) 14:e0226361. 10.1371/journal.pone.0226361 31830138 PMC6907772

[25] Schneidman ES. The psychological autopsy. Suicide Life Threat Behav. (1981) 11:325–40. 10.1111/j.1943-278X.1981.tb01009.x

[26] NHC. Chinese Health Statistics Yearbook 2019. China union medical college press. (2019).

[27] Shandong Provincial Bureau of Statistics. Shandong Statistical Yearbook. China Statistics Press. (2019).

[28] Sun L Zhou C. Association between body mass index and suicidal ideation among seniors in Shandong, China. Compr Psychiatry. (2018) 82:68–72. 10.1016/j.comppsych.2018.01.008 29407361

[29] Nock MK Green JG Hwang I McLaughlin KA Sampson NA Zaslavsky AM. Prevalence, correlates, and treatment of lifetime suicidal behavior among adolescents: results from the National Comorbidity Survey Replication Adolescent Supplement. JAMA psychiatry. (2013) 70:300–10. 10.1001/2013.jamapsychiatry.55 23303463 PMC3886236

[30] Kalmbach DA Arnedt JT Song PX Guille C Sen S. Sleep disturbance and short sleep as risk factors for depression and perceived medical errors in first-year residents. Sleep. (2017) 40:zsw073. 10.1093/sleep/zsw073 28369654 PMC6084763

[31] Trockel MT Menon NK Rowe SG Stewart MT Smith R Lu M. Assessment of physician sleep and wellness, burnout, and clinically significant medical errors. JAMA network open. (2020) 3:e2028111. 10.1001/jamanetworkopen.2020.28111 33284339 PMC12064096

[32] Shanafelt TD Dyrbye LN West CP Sinsky C Tutty M Carlasare LE. Suicidal ideation and attitudes regarding help seeking in US physicians relative to the US working population. Mayo Clinic Proc. (2021) 96:2067–80. 10.1016/j.mayocp.2021.01.033 34301399

[33] Milette K Hudson M Baron M Thombs BD. Comparison of the PHQ-9 and CES-D depression scales in systemic sclerosis: internal consistency reliability, convergent validity and clinical correlates. Rheumatology. (2010) 49:789–96. 10.1093/rheumatology/kep443 20100794

[34] Thombs BD Hudson M Schieir O Taillefer SS Baron M. Reliability and validity of the center for epidemiologic studies depression scale in patients with systemic sclerosis. Arthritis Rheum. (2008) 59:438–43. 10.1002/art.23329 18311754

[35] Jiang L Wang Y Zhang Y Li R Wu H Li C. The reliability and validity of the center for epidemiologic studies depression scale (CES-D) for Chinese University Students. Front Psychiatry. (2019) 10:315. 10.3389/fpsyt.2019.00315 31178764 PMC6537885

[36] Bolin JH. Introduction to mediation, moderation, and conditional process analysis: a regression-based approach. J Educ Meas. (2014) 51:335–7. 10.1111/jedm.12050

[37] Arnold J Tango J Walker I Waranch C McKamie J Poonja Z. An evidence-based, longitudinal curriculum for resident physician wellness: the 2017 resident wellness consensus summit. West J Emerg Med. (2018) 19:337–41. 10.5811/westjem.2017.12.36244 29560063 PMC5851508

[38] Elkbuli A Sutherland M Shepherd A Kinslow K Liu H Ang D. Factors influencing US physician and surgeon suicide rates 2003-2017: analysis of the CDC-national violent death reporting system. Ann Surg. (2020). 10.1097/SLA.0000000000004575 33156059

[39] Braquehais MD González-Irizar O Nieva G Mozo X Llavayol E Pujol T. Assessing high risk of suicide amongst physicians and nurses in treatment. Psychiatry Res. (2020) 291:113237. 10.1016/j.psychres.2020.113237 32619824

[40] Menon NK Shanafelt TD Sinsky CA Linzer M Carlasare L Brady KJS. Association of physician burnout with suicidal ideation and medical errors. JAMA Netw Open. (2020) 3:e2028780. 10.1001/jamanetworkopen.2020.28780 33295977 PMC7726631

[41] Tawfik DS Profit J Morgenthaler TI Satele DV Sinsky CA Dyrbye LN. Physician burnout, well-being, and work unit safety grades in relationship to reported medical errors. Mayo Clinic Proc. (2018) 93:1571–80. 10.1016/j.mayocp.2018.05.014 30001832 PMC6258067

[42] Mann JJ. A current perspective of suicide and attempted suicide. Ann Intern Med. (2002) 136:302–11. 10.7326/0003-4819-136-4-200202190-00010 11848728

[43] Waterman AD Garbutt J Hazel E Dunagan WC Levinson W Fraser VJ. The emotional impact of medical errors on practicing physicians in the United States and Canada. Joint Commission J Quality Patient Safety. (2007) 33:467–76. 10.1016/S1553-7250(07)33050-X 17724943

[44] Center C Davis M Detre T Ford DE Hansbrough W Hendin H. Confronting depression and suicide in physicians: a consensus statement. Jama. (2003) 289:3161–6. 10.1001/jama.289.23.3161 12813122

[45] West CP Huschka MM Novotny PJ Sloan JA Kolars JC Habermann TM. Association of perceived medical errors with resident distress and empathy: a prospective longitudinal study. Jama. (2006) 296:1071–8. 10.1001/jama.296.9.1071 16954486

[46] Christensen JF Levinson W Dunn PM. The heart of darkness: the impact of perceived mistakes on physicians. J Gen Intern Med. (1992) 7:424–31. 10.1007/BF02599161 1506949

[47] American College of Emergency Physicians. Disclosure of medical errors. Ann Emerg Med. (2017) 70:121–2. 10.1016/j.annemergmed.2017.04.045 28645408

[48] Rodziewicz TL Houseman B Hipskind JE. Medical Error Reduction and Prevention. StatPearls Treasure Island (FL): StatPearls Publishing. (2021). 29763131

[49] Zapata Roblyer MI Betancourth Zambrano S. Crime victimization and suicidal ideation among colombian college students: the role of depressive symptoms, familism, and social support. J Interpers Violence. (2020) 35:1367–88. 10.1177/0886260517696856 29294673

[50] Schnell T Gerstner R Krampe H. Crisis of meaning predicts suicidality in youth independently of depression. Crisis. (2018) 39:294–303. 10.1027/0227-5910/a000503 29473473

[51] Nelson C Cyr KS Corbett B Hurley E Gifford S Elhai JD. Predictors of posttraumatic stress disorder, depression, and suicidal ideation among Canadian Forces personnel in a National Canadian Military Health Survey. J Psychiatr Res. (2011) 45:1483–8. 10.1016/j.jpsychires.2011.06.014 21752395

[52] Lu L Xu L Luan X Sun L Li J Qin W. Gender difference in suicidal ideation and related factors among rural elderly: a cross-sectional study in Shandong, China. Ann Gen Psychiatry. (2020) 19:2. 10.1186/s12991-019-0256-0 31956335 PMC6958769

[53] Chang Q Yip PSF Chen YY. Gender inequality and suicide gender ratios in the world. J Affect Disord. (2019) 243:297–304. 10.1016/j.jad.2018.09.032 30261445

[54] Nishi D Susukida R Kuroda N Wilcox HC. The association of personal importance of religion and religious service attendance with suicidal ideation by age group in the National Survey on Drug Use and Health. Psychiatry Res. (2017) 255:321–7. 10.1016/j.psychres.2017.06.007 28601715

[55] Lawrence RE Oquendo MA Stanley B. Religion and suicide risk: a systematic review. Arch Suic Res. (2016) 20:1–21. 10.1080/13811118.2015.1004494 26192968 PMC7310534

[56] Lin W Wang H Gong L Lai G Zhao X Ding H. Work stress, family stress, and suicide ideation: A cross-sectional survey among working women in Shenzhen, China. J Affect Disord. (2020) 277:747–54. 10.1016/j.jad.2020.08.081 32919296

